# Awareness of post-transplant endocrine disorders among kidney transplant clinicians: results of an Italian survey

**DOI:** 10.1007/s40618-025-02741-y

**Published:** 2025-10-29

**Authors:** Bianca Pellegrini, Vincenzo Cantaluppi, Gianluca Aimaretti, Mariano Ferraresso, Jacopo Romagnoli, Cristina Silvestre, Giorgia Comai, Umberto Maggiore, Francesca Leone, Rosita Greco, Marcello Maggiolini, Michele Provenzano, Gianluigi Zaza

**Affiliations:** 1 Department of Pharmacy, Health and Nutritional Sciences, University of Calabria, 87036 Rende, Italy; 2 Nephrology and Kidney Transplantation Unit, Department of Translational Medicine (DIMET), University of Piemonte Orientale (UPO), “Maggiore della Carità” University Hospital, Novara, Italy; 3 Endocrinology, Department of Translational Medicine, Università del Piemonte Orientale, Novara, Italy; 4 General Surgery and Kidney Transplantation, Fondazione IRCCS Ca’ Granda Ospedale Maggiore Policlinico, Milan, Italy; 5 Department of Clinical Sciences and Community Health, Università degli Studi di Milano, Milan, Italy; 6 Renal Transplant Unit, Fondazione Policlinico Universitario A. Gemelli IRCCS, Rome, Italy; 7 Kidney and Pancreas Transplantation Unit, Azienda Ospedaliera Universitaria di Padova, Padova, Italy; 8 Nephrology, Dialysis and Kidney Transplant Unit, IRCCS-Azienda Ospedaliero-Universitaria di Bologna, Bologna, Italy; 9 Department of Medical and Surgical Sciences (DIMEC), Alma Mater Studiorum-University of Bologna, Bologna, Italy; 10 Nephrology Unit, University Hospital of Parma, Parma, Italy; 11 Nephrology, Dialysis and Transplant Unit, “SS. Annunziata” Hospital, Cosenza, Italy

**Keywords:** Kidney transplantation, Endocrine disorders, Bone disease, Thyroid disorders, Pituitary disorders, Adrenal disorders

## Abstract

**Purpose:**

Endocrine disorders, which are commonly associated with End-Stage Kidney Disease, may either persist or emerge *de novo* in the post-transplant period. Despite their clinical relevance, the literature remains limited, and current guidelines offer only vague recommendations regarding the diagnosis and treatment.

**Methods:**

A 44-item survey was sent via email to each kidney transplant center to assess transplant physicians’ interest in the endocrine disorders of kidney transplant recipients (KTRs) undergoing regular follow-up. The questionnaire was composed of 6 sections: general information; bone disease; thyroid disorders; pituitary disorders; adrenal disorders; gonadal disorders, fertility, and sexuality.

**Results:**

Out of the 41 centers, 29 transplant physicians participated in the study (70.7%). The prevalence of osteoporosis was greater than 50% in KTRs while the prevalence of fractures is likely underestimated, as most centers practice routine bone mineral density (BMD) screening through DEXA scan but do not routinely perform spinal radiography to detect vertebral deformities. The kidney transplant clinicians routinely assess thyroid hormone levels as part of clinical history although the timing varies widely among centers. Adrenal function is not routinely assessed during follow-up in a substantial number of centers, with only 14% conducting regular biochemical evaluations. Many centers show insufficient interest in investigating gonadal function, with 20% not addressing it during clinical history taking and 35% unaware of the incidence of menstrual irregularities or erectile dysfunction in their KTRs.

****Conclusions**:**

The survey revealed significant variability in the management of endocrine disorders across the Italian transplant centers. The development of guidelines for early detection and management would significantly improve the individualized care of this fragile patient population.

**Supplementary Information:**

The online version contains supplementary material available at 10.1007/s40618-025-02741-y.

## Introduction

Kidney transplantation is the gold standard treatment for patients with advanced chronic kidney disease. Despite significant improvements in early graft survival due to advancements in graft preservation, surgical techniques, and the management and personalization of immunosuppressive protocols, long-term allograft survival remains suboptimal, and the rate of comorbidities remains high [1]. Among these, endocrine disorders may play a significant role.

These conditions may result from uremia and chronic inflammation associated with end-stage kidney disease (ESKD) [2], as well as from the biological changes that occur after transplantation. Following transplantation, the chronic use of immunosuppressive medications, particularly calcineurin inhibitors (e.g., cyclosporine, tacrolimus), can contribute to the onset and progression of endocrinopathies and endocrine gland malignancies [3, 4]. In addition, long-term glucocorticoid use can dysregulate key hormonal axes and lead to systemic complications associated with adrenal insufficiency [5].

The most frequently described endocrine disorders in kidney transplant recipients (KTRs) are dysthyroidism (including subclinical or overt hypothyroidism and impaired T4 to T3 conversion) [6], adrenal insufficiency [7], and primary hypogonadism [8]. If left untreated, these conditions can adversely affect metabolism, impair graft function [9–11], increase cardiovascular and mortality risk [12–15]. Moreover, reproductive and sexual dysfunctions, including altered spermatogenesis, menstrual irregularities, infertility, and erectile dysfunction, are common in KTRs [16–18]. These disorders can significantly impact patients’ quality of life, and the role of immunosuppressive drugs in their pathogenesis is not fully understood. They are becoming an increasingly important focus in transplantation medicine.

Additionally, while speculative, studies suggest a correlation between FT3 levels and serum creatinine post-transplantation [10, 11], as well as a potential role for prolactin in modulating the immune response [19, 20] that underlies graft rejection.

KTRs have increased risk for chronic kidney disease-mineral and bone disease (CKD-MBD) with a high prevalence of hypophosphatemia, hypercalcemia, and hypovitaminosis D [21] which are associated with adverse clinical outcomes [22–26].

Chronic exposure to glucocorticoids, even at low doses, may also lead to systemic comorbidities such as metabolic syndrome [27] and hypertension [28]. Prolonged glucocorticoid use can suppress the hypothalamic-pituitary-adrenal axis, increasing the risk of adrenal insufficiency under stress conditions or intercurrent illness [29].

Despite the frequent occurrence of these endocrine disorders in KTRs, studies on large populations assessing their prevalence and impact are still lacking, reflecting the limited attention clinicians have given to this important issue. Furthermore, due to the scarcity of evidence, the current guidelines offer few clear recommendations for the diagnosis and treatment of endocrine disorders in KTRs. The approach to these disorders often varies depending on the awareness and interest of the clinicians overseeing the KTR follow-up.

Therefore, the aim of this survey is to assess the awareness and approach of Italian transplant centers toward the endocrine disorders affecting KTRs.

## Methods

### Organizing committee and participants

The survey was designed by the joint committee of the Italian Society of Nephrology. In Italy, kidney transplantation programs (*n* = 41) are part of the public health system, and all kidney transplant centers were invited to participate.

### Survey

A 44-item survey was sent via email to each participant, inquiring about the interest of kidney transplant physicians in the endocrine disorders of kidney transplant recipients undergoing regular follow-up. All responses were kept confidential and were evaluated accordingly. The survey was divided into 6 sections, each addressing specific topics related to clinical and technical aspects. The survey gathered both physicians’ opinions and aggregate data on patients from each transplant center. A designated respondent from each center completed a single questionnaire to avoid duplicate responses. Responses remained anonymous. Data collected from the responses were analyzed by two researchers from the Department of Pharmacy, Health and Nutritional Sciences, University of Calabria, Italy.

## Results

### General section (S.1)

**Fig. 1:**
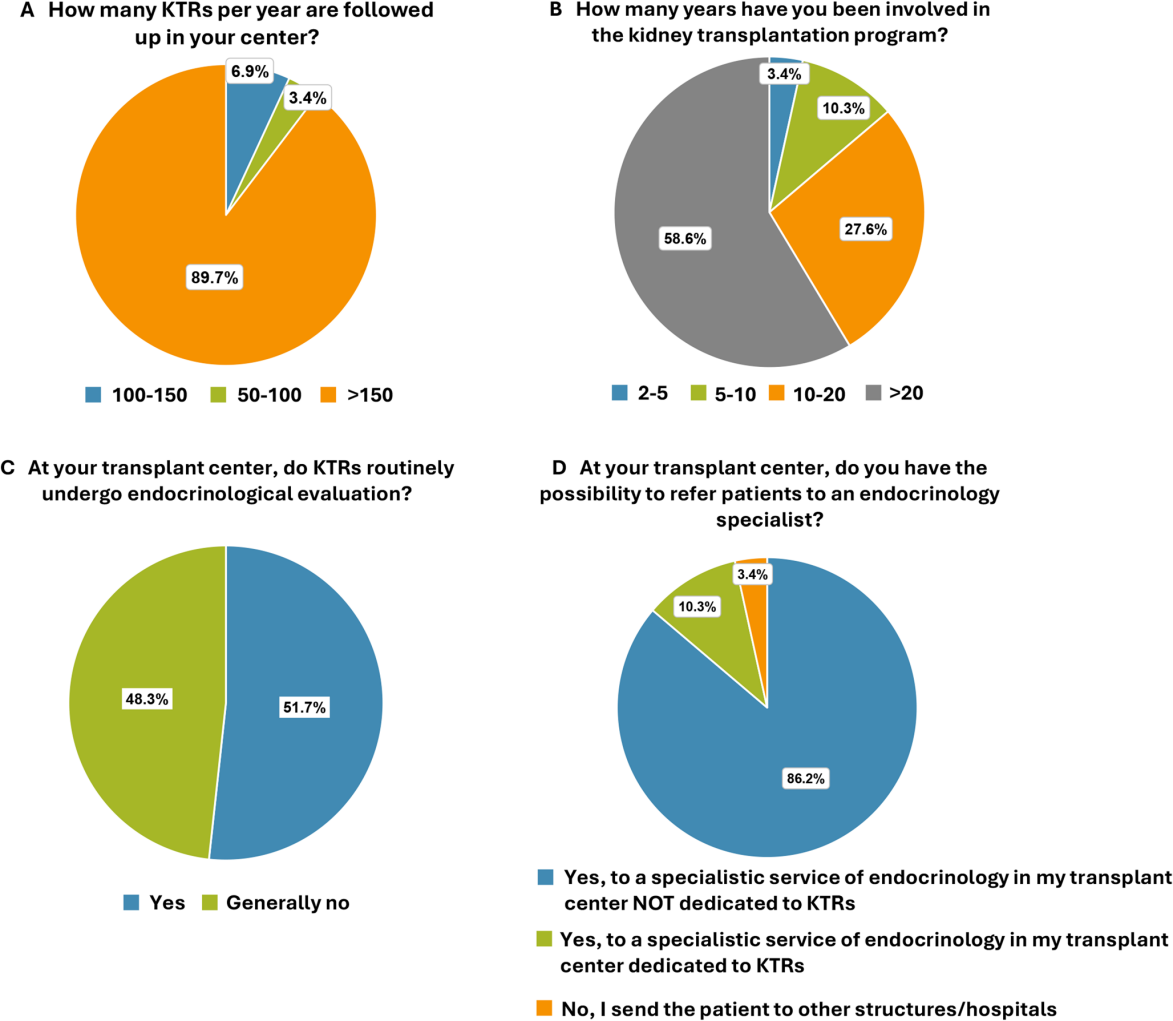
Survey participant characteristics and endocrinological evaluation.** (A)** Number of kidney transplant recipients followed up/year. **(B)** Experience (in years) of transplant physicians in kidney transplantation. **(C)** Routine endocrinological evaluation of KTRs and **(D)** where they can be evaluated

Out of the 41 centers, 29 transplant physicians participated in the study (70.7%). Among these, 26/29 centers (89.7%) reported a regular follow-up of > 150 KTRs/year, 2/29 (6.9%) followed 100–150 KTRs/year, and 1/29 (3.4%) followed 50–100 KTRs/year (Fig. 1A).

In terms of experience, 17/29 participants (58.6%) had more than 20 years of experience in kidney transplantation, 8/29 (27.6%) had between 10 and 20 years, 3/29 (10.3%) had 5 to 10 years, and 1/29 (3.4%) had less than 5 years of experience (Fig. 1B).

In 15/29 (51.7%) centers, KTRs were routinely evaluated by an endocrinologist, regardless of their clinical history of endocrine disorders (Fig. 1C). Only 3/29 centers (10.3%) had a dedicated transplant endocrinology service, whereas 25/29 centers (86.2%) used a general intra-hospital endocrinology service, and 1/29 (3.4%) referred patients to other hospitals (Fig. 1D).

## Specific sections

### Bone disease (S.2)

#### Epidemiology

**Fig. 2:**
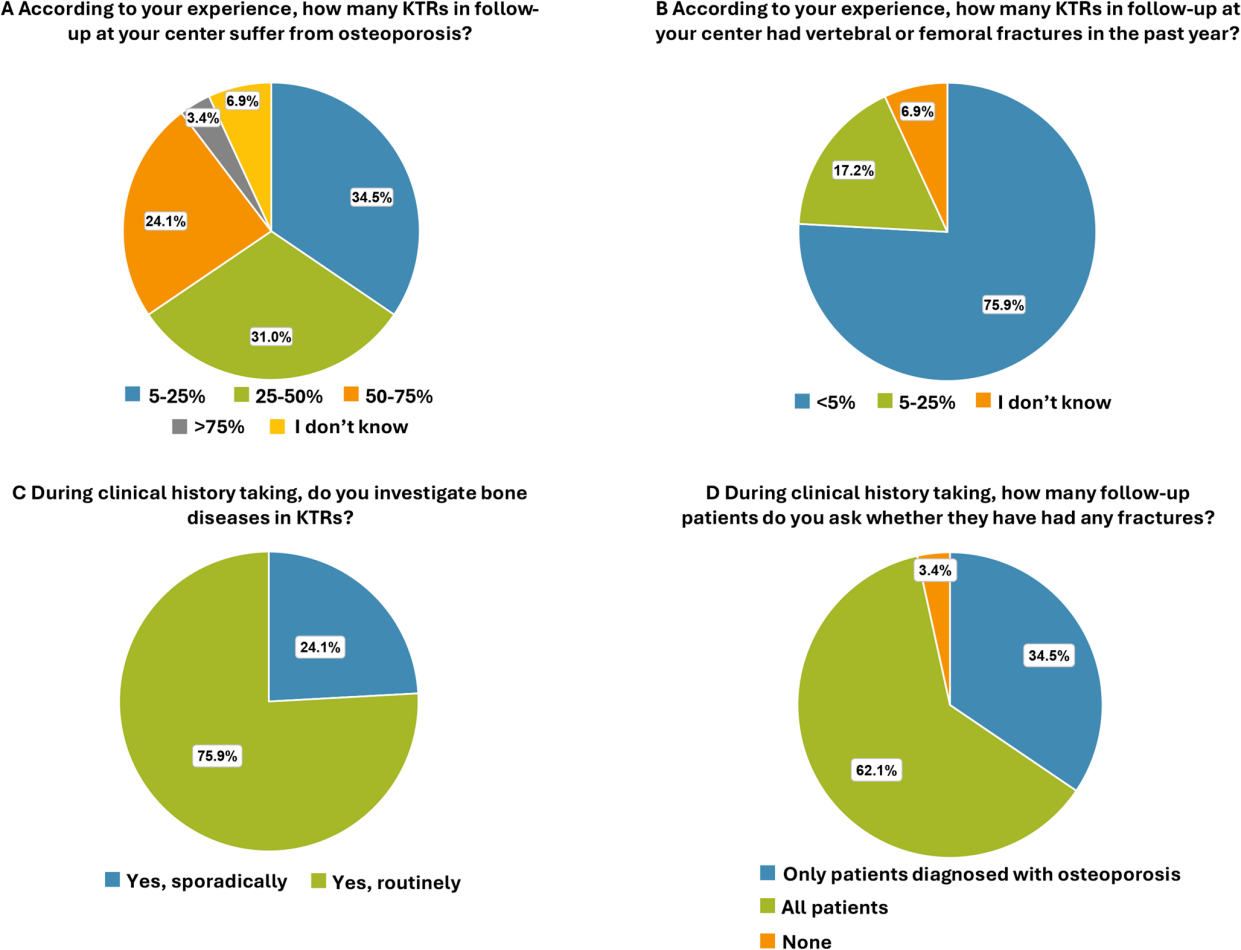
Bone disease.** (A)** Prevalence of KTRs suffering from osteoporosis in the past year. **(B)** Prevalence of KTRs with vertebral/femoral fractures in the past year. **(C)** Frequency of assessing bone diseases during clinical history taking. **(D)** Number of patients asked about previous fractures as part of clinical history

Of the 29 centers, 10 (34.5%) estimated the prevalence of osteoporosis [diagnosed as low bone density (BMD) at dual energy X-ray absorptiometry (DEXA) scan] in KTRs to be between 5% and 25%; 9 centers (31.0%) estimated it to be between 25% and 50%; 7 centers (24.1%) estimated a prevalence of 50% to 75%; and 1 center (3.4%) estimated it to be greater than 75%. Additionally, 2/29 (6.9%) centers were unable to estimate the prevalence (Fig. 2A).

The prevalence of vertebral/femoral fractures was estimated to be < 5% by 22 physicians (75.9%), and 5–25% by 5 (17.2%) in the past year, while 2 (6.9%) were unable to estimate this data (Fig. 2B).

### Clinical history

Of the 29 physicians involved in the follow-up of KTRs, 22 (75.9%) routinely assessed for bone diseases during clinical history taking (conducted through verbal questions or written questionnaires addressing prior fractures, bone pain, and classical risk factors, using simple and accessible language), while 7 (24.1%) do it sporadically (Fig. 2C). In particular, 18 (62.1%) always ask about fractures in all KTRs, 10 (34.5%) inquired only for patients diagnosed with osteoporosis, and 1 (3.4%) do not ask about bone diseases (Fig. 2D).

### Laboratory and instrumental evaluations

**Fig. 3:**
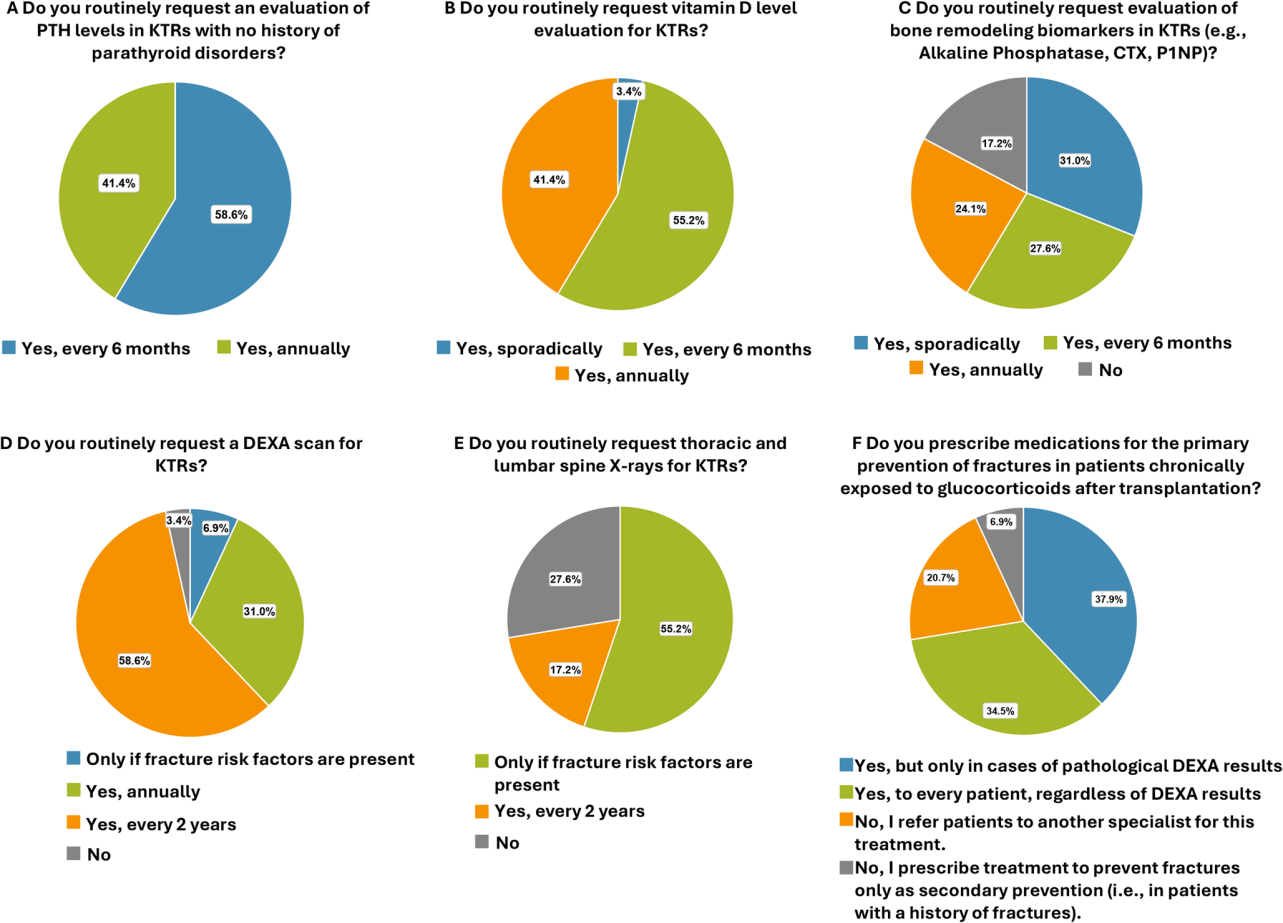
Monitoring parathyroid and bone health. Frequency of assessment of **(A)** PTH, **(B)** vitamin D, and **(C)** bone remodeling biomarker levels. Frequency of request of **(D)** DEXA scan and **(E)** thoracic and lumbar spine X-rays. **(F)** Prescription of drugs to prevent fractures in patients chronically exposed to glucocorticoids

PTH levels are evaluated every 6 months in 17/29 centers (58.6%) and annually in 12/29 centers (41.4%) (Fig. 3A). Vitamin D levels are evaluated every 6 months in 16/29 centers (55.2%) and annually in 12/29 centers (41.4%) (Fig. 3B). Bone remodeling biomarkers (e.g., alkaline phosphatase, CTX, P1NP) are sporadically assessed in 9 centers (31.0%), every 6 months in 8 (27.6%), and annually in 7 centers (24.1%) (Fig. 3C). By contrast, 5 centers (17.2%) do not evaluate bone remodeling biomarkers at all. DEXA scan is requested annually in 9/29 centers (31.0%) and every 2 years in 17/29 centers (58.6%) for all patients, while 2/29 (6.9%) request it only for high-risk patients. In addition, 1/29 center (3.4%) do not request DEXA scan at all (Fig. 3D). Thoracic and lumbar spine X-rays are requested every 2 years in 5 centers (17.2%), only for high-risk patients in 16 centers (55.2%), and not requested at all in 8 centers (27.6%) (Fig. 3E).

### Treatment

In 21 centers (72.4%), the staff physicians prescribe drugs for primary prevention of fractures in KTRs chronically exposed to glucocorticoids after transplantation: 10 (34.5%) regardless of DEXA results, and 11/29 (37.9%) based on pathological DEXA results. In 6/29 centers (20.7%), treatment is not provided, and patients are referred to specialists, including endocrinologists (5) and rheumatologists (1), while 2 centers (6.9%) prescribe the treatment only as secondary fractures prevention (Fig. 3F).

### Thyroid disorders (S.3)

#### Epidemiology

**Fig. 4:**
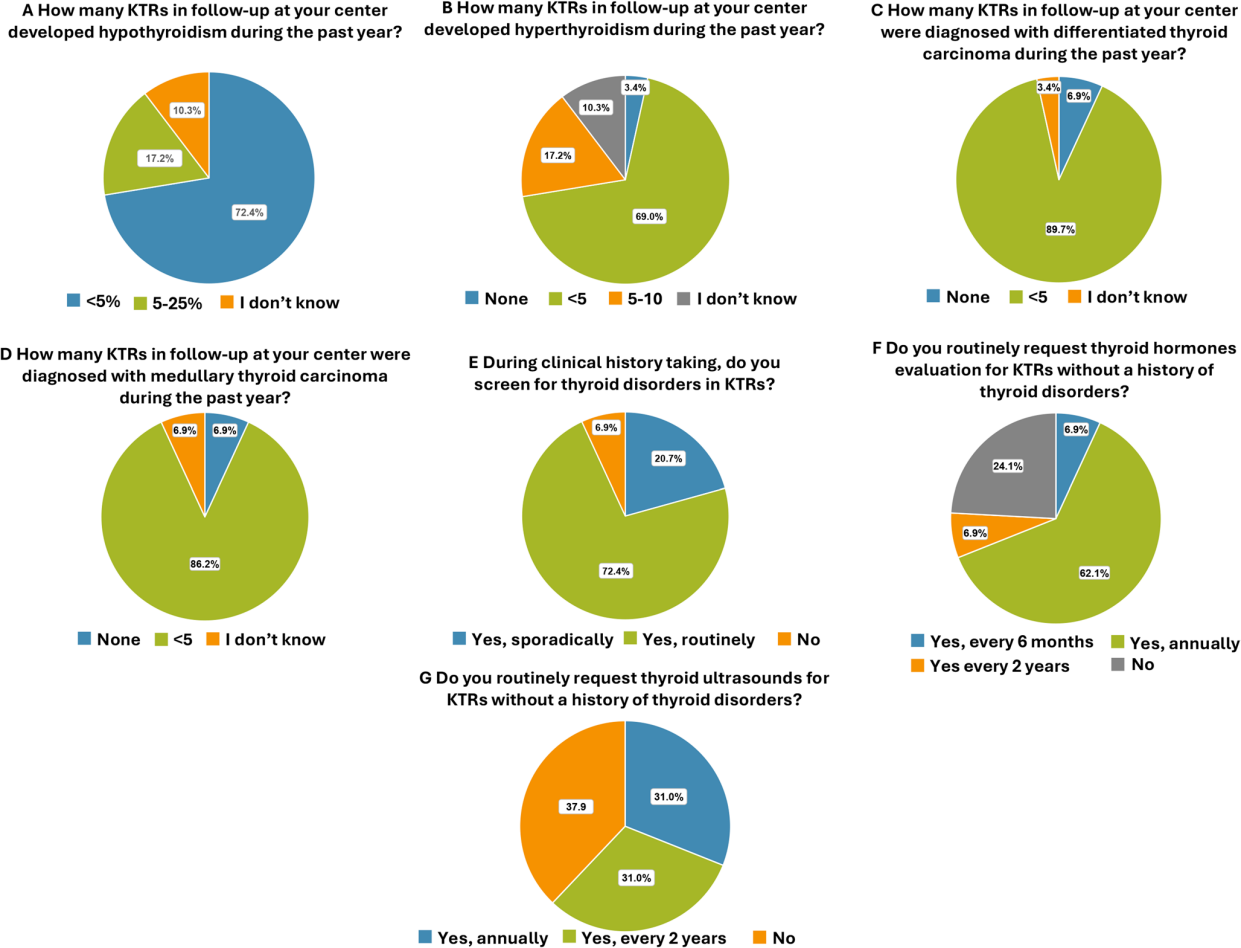
Thyroid disorders. Prevalence of KTRs who developed: **(A)** hypothyroidism, **(B)** hyperthyroidism, **(C)** differentiated thyroid carcinoma, **(D)** medullary thyroid carcinoma in the past year. Frequency of assessment of **(E)** thyroid disorders and **(F)** thyroid hormones during clinical history taking. **(G)** Frequency of request of ultrasound of the thyroid

The prevalence of hypothyroidism was reported as < 5% by 21 centers (72.4%), and 5–25% by 5 centers (17.2%) in the past year. Three centers (10.3%) lacked data (Fig. 4A).

The prevalence of hyperthyroidism was < 5 cases annually in 20 centers (69.0%), 5–10 cases in 5 (17.2%) in the past year, and no cases in 1 center (3.4%). Data were unavailable in 3 centers (10.3%) (Fig. 4B).

Regarding differentiated thyroid carcinomas, 26 centers (89.7%) registered fewer than five new cases in the past year, while 2 centers (6.9%) did not report any new cases, and in 1 center (3.4%) this data was missing (Fig. 4C).

The diagnosis of medullary thyroid carcinomas was < 5 in 25 centers (86.2%), with no new cases in 2 centers (6.9%), while data was missing in 2 centers (6.9%) (Fig. 4D).

### Clinical history

Among the 29 centers evaluated, 21 (72.4%) routinely assess thyroid disorders during clinical history taking (conducted through verbal questions or written questionnaires and a thorough review of available clinical documents also from other specialists), 6 (20.7%) only sporadically, and 2 (6.9%) do not inquire about thyroid history at all (Fig. 4E).

### Laboratory and instrumental evaluations

In 22 centers (75.9%), thyroid hormones are routinely measured during post-transplant follow-up. In particular, 2/29 (6.9%) measure them every 6 months, 18/29 (62.1%) annually, and 2/29 (6.9%) every 2 years whereas 7 centers (24.1%) do not request the evaluation of these hormones at all (Fig. 4F).

In 18/29 centers (62.0%) thyroid ultrasounds are performed: 9 (31.0%) annually, 9 (31.0%) every 2 years, while 11 centers (37.9%) do not perform thyroid ultrasounds during follow-up (Fig. 4G).

### Pituitary disorders (S.4)

#### Epidemiology

**Fig. 5:**
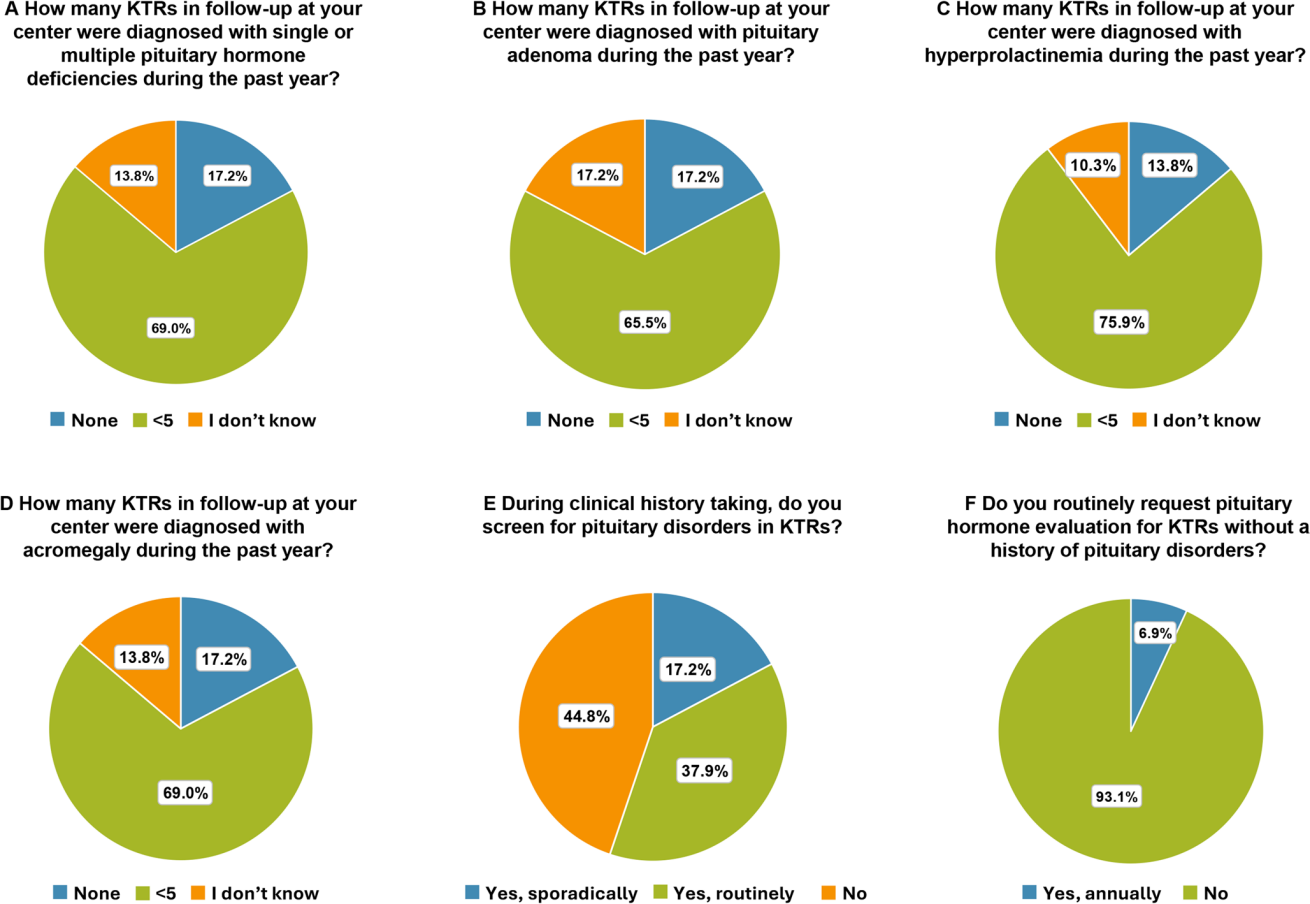
Pituitary disorders. Prevalence of KTRs who developed: **(A)** single or multiple pituitary hormone deficiencies, **(B)** pituitary adenoma, **(C)** hyperprolactinemia, **(D)** acromegaly in the past year. Frequency of assessment of **(E)** pituitary disorders and **(F)** pituitary hormones during clinical history taking

Among the 29 centers involved, 5 (17.2%) reported no new diagnoses of single/multiple pituitary deficiency while 20 (69.0%) reported fewer than 5 new cases in the last year and 4 centers (13.8%) lacked data (Fig. 5A).

Moreover, 5 (17.2%) reported no new diagnoses of pituitary adenoma, while 19/29 (65.5%) reported fewer than 5 new cases in the last year (Fig. 5B).

In 22 centers (75.9%), fewer than 5 new cases of hyperprolactinemia were reported, and 4 (13.8%) reported no new cases (Fig. 5C).

The prevalence of acromegaly was < 5 cases in 20 centers (69.0%), no cases in 5 centers (17.2%), and 4 centers (13.8%) lacked data (Fig. 5D).

### Clinical history

In 11/29 centers (37.9%) pituitary disorders are routinely assessed as part of clinical history (conducted through verbal questions or written questionnaires and a thorough review of available clinical documents also from other specialists), 5/29 (17.2%) do so sporadically, and 13/29 centers (44.8%) do not inquire about pituitary disorders at all (Fig. 5E).

### Laboratory and instrumental evaluations

Only 2 of 29 centers (6.9%) perform pituitary hormone evaluations annually, while the remaining centers do not conduct these tests (Fig. 5F).

### Adrenal disorders (S.5)

#### Epidemiology

**Fig. 6:**
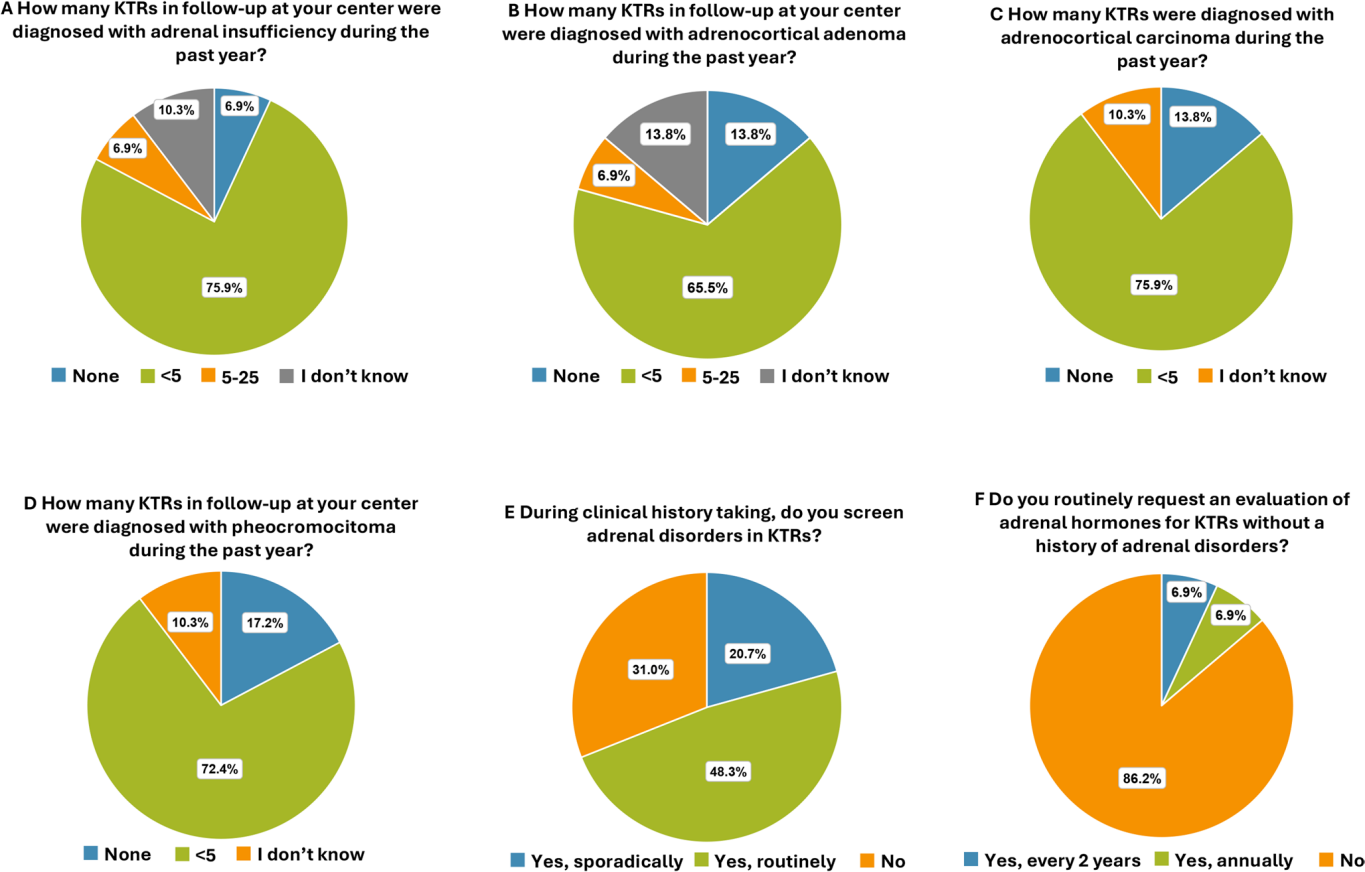
Adrenal disorders. Prevalence of KTRs who developed: **(A)** adrenal insufficiency **(B)** adrenocortical adenoma, **(C)** adrenocortical carcinoma and **(D)** pheochromocytoma in the past year. Frequency of assessment of **(E)** adrenal disorder and **(F)** adrenal hormones during clinical history taking

Only 2 centers (6.9%) reported no new diagnoses of adrenal insufficiency, while 22 (75.9%) reported < 5 new cases in the last year and 2 centers (6.9%) documented between 5 and 25 cases (Fig. 6A).

Fewer than five new cases of adrenocortical adenoma were reported in 19 of the 29 centers (65.5%), between 5 and 25 cases in 2 centers (6.9%), while no new cases of adrenocortical adenoma were reported in 4 centers (13.8%) (Fig. 6B).

The prevalence of adrenocortical carcinoma was < 5 cases in 22/29 centers (75.9%) and was absent in 13.8% (Fig. 6C).

The prevalence of pheochromocytoma was < 5 cases in 21 centers (72.4%) and was absent in 5 (17.2%) (Fig. 6D).

### Clinical history

The majority of respondents (48.3%) routinely inquire about adrenal disorders during clinical history taking (conducted through verbal questions or written questionnaires and a thorough review of available clinical documents also from other specialists), while 6/29 (20.7%) do so sporadically and 9/29 centers (31.0%) do not inquire about adrenal disorders at all (Fig. 6E).

### Laboratory and instrumental evaluations

Only 4/29 centers perform regular biochemical evaluations of adrenal hormones: 2/29 centers (6.9%) every 2 years, and 2/29 centers (6.9%) annually (Fig. 6F).

### Gonadal disorders, fertility, and sexuality (S.6)

#### Epidemiology

**Fig. 7:**
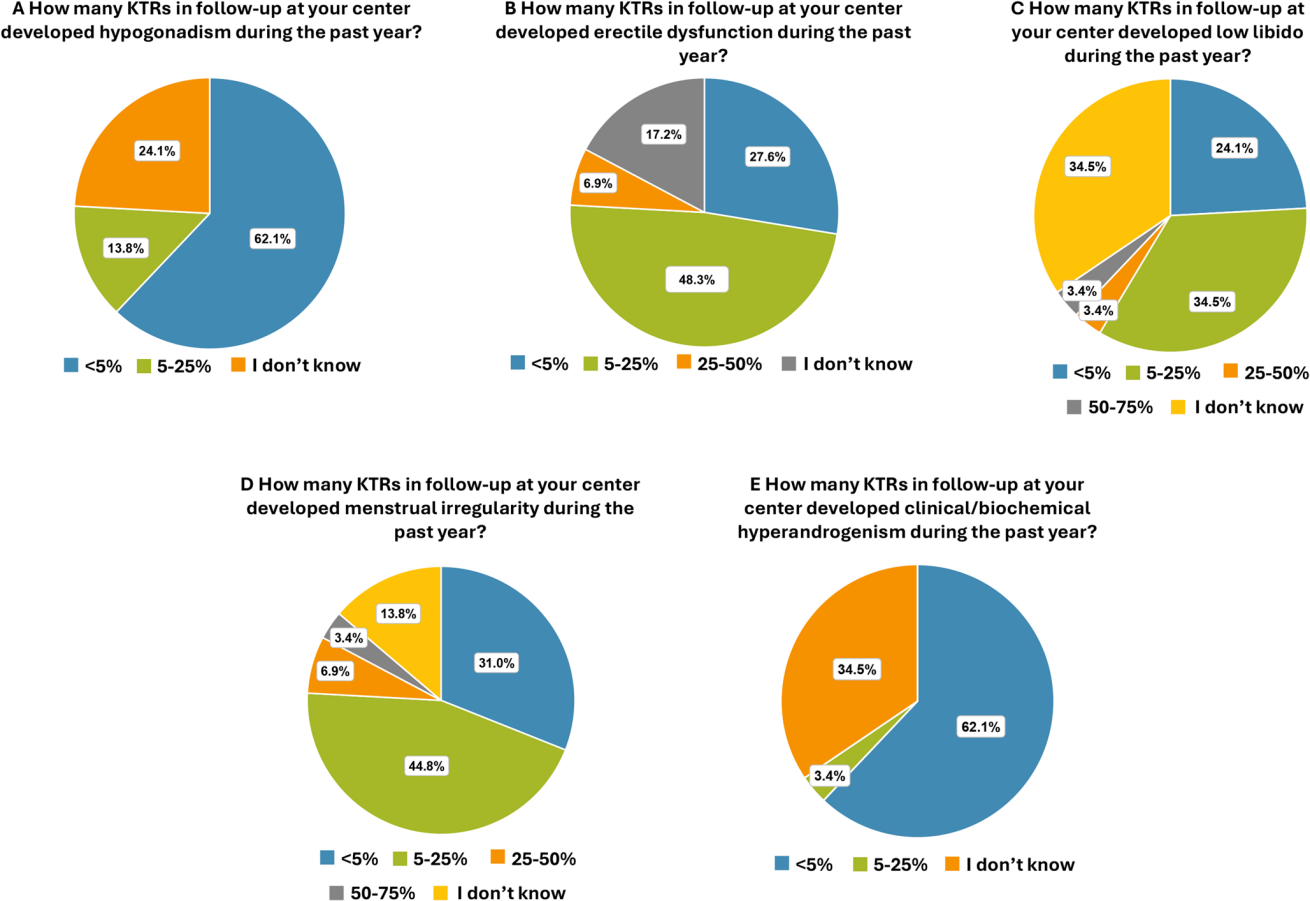
Gonadal disorders, fertility, and sexuality. Prevalence of KTRs who developed: **(A)** hypogonadism, **(B)** erectile dysfunction, **(C)** low libido **(D)** menstrual irregularity and **(E)** clinical/biochemical hyperandrogenism in the past year

In 18 centers (62.1%), it was reported that fewer than 5% of KTRs developed hypogonadism in the past year. In 4 centers (13.8%), the reported prevalence ranged from 5% to 25% of KTRs (Fig. 7A).

Fewer than 5% of the KTRs in the follow-up developed erectile dysfunction in 8 centers (27.6%), between 5% and 25% in 14 centers (48.3%), and between 25% and 50% in 2 centers (6.9%) (Fig. 7B).

In the past year, 7 centers (24.1%) reported new cases of low libido in < 5% of KTRs; 10 centers (34.5%) reported a prevalence of 5–25%; and 1 center (3.4%) reported a prevalence of 25–50%; 1 center (3.4%) documented a prevalence between 50% and 75% (Fig. 7C).

Menstrual irregularities could not be estimated in 4 centers (13.8%), while 9 centers (31.0%) reported that < 5% of KTRs developed menstrual irregularities during the past year; 13 centers (44.8%) reported that they occurred in 5–25% of KTRs (Fig. 7D). In the past year, the prevalence of clinical/biochemical hyperandrogenism was < 5% in 18 centers (62.1%) and between 5% and 25% in 1 center (3.4%) (Fig. 7E).

### Clinical history

**Fig. 8:**
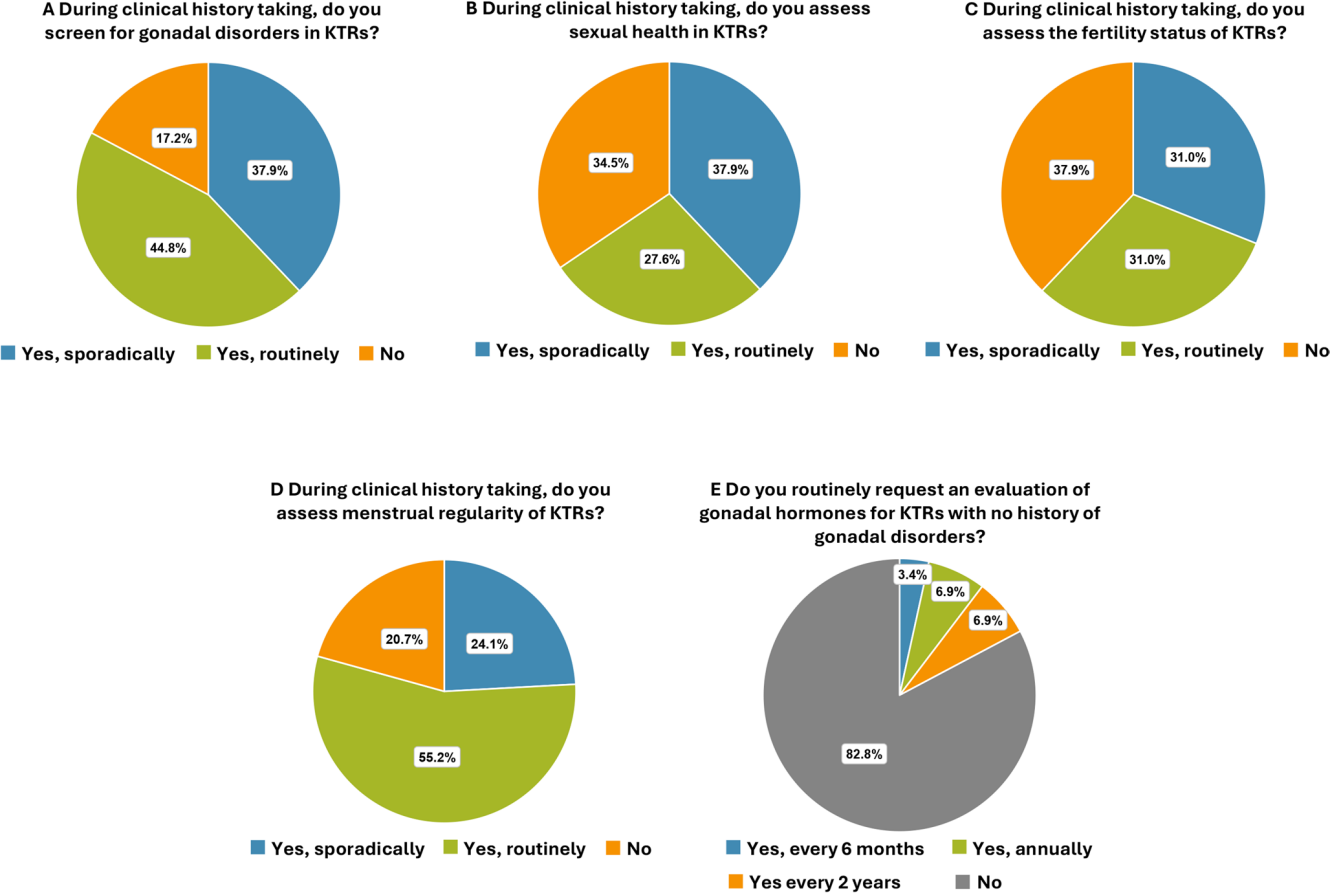
Gonadal and sexual health assessment during clinical history taking. Frequency of assessment of **(A)** gonadal disorders, **(B)** sexual health, **(C)** fertility status, **(D)** menstrual regularity, and **(E)** gonadal hormones during clinical history taking

The physicians involved in the follow-up of KTRs reported that, during collection of clinical history (conducted through verbal questions or written questionnaires and a thorough review of available clinical documents also from other specialists), they do not investigate gonadal disorders in 5 centers (17.2%) (Fig. 8A), sexual health in 10 centers (34.5%) (Fig. 8B), fertility in 11 centers (37.9%) (Fig. 8C), and menstrual regularity in 6 centers (20.7%) (Fig. 8D).

### Laboratory and instrumental evaluations

Gonadal hormones are routinely assessed in 5 centers: every 6 months in 1 center (3.4%), annually in 2 centers (6.9%) and every 2 years in 2 centers (6.9%) (Fig. 8E).

## Discussion

To date, there are no surveys in the literature that investigate the interest of physicians involved in the follow-up of kidney transplant recipients (KTRs), in endocrine disorders in these patients. This survey aimed to gather information on the clinical approaches and the level of awareness among Italian transplant centers regarding the critical issue of endocrine disorders. The participants were experienced physicians from high-volume centers with expertise in kidney transplantation.

The level of interest in endocrine disorders and access to endocrinological evaluations among Italian transplant centers is highly heterogeneous. Half of the centers routinely provide a specialist evaluation by an endocrinologist for KTRs as part of their follow-up care, but only a few centers have dedicated endocrinology services for these complex and fragile patients.

After transplantation, CKD-related hyperparathyroidism is expected to normalize, yet elevated parathyroid hormone (PTH) levels persist in about 50% of KTRs [30–32]. In addition to CKD-related bone disease and hyperparathyroidism, immunosuppressive drugs, particularly cyclosporine (CSA) and glucocorticoids, together with vitamin D deficiency contribute to bone fragility and the high risk of fractures, even when glomerular filtration is restored [26, 33, 34]. Bone mineral density (BMD) decreases during the first few years after transplantation before stabilizing in the long term [35]. KTRs experience a vertebral fractures incidence between 7.2 and 15.4 per 1000 patient-years [36–38] detected through imaging such as vertebral morphometry or MRI.

Damage to bone microarchitecture increases fracture risk in kidney transplant recipients (KTRs), even in those with normal or only mildly reduced BMD. Bisphosphonates, although improving BMD at vertebral and femoral levels, have uncertain effects on vertebral fracture reduction risk [39]. Based on lumbar and femoral T-score evaluation via DEXA scan, the rate of osteoporosis ranged from 11% to 53% of KTRs and osteopenia ranged from 7% to 52.5% [21, 40–47]. However, these data likely underestimate the true burden of bone disease in KTRs, suggesting that periodic DEXA scan evaluations alone may not be sufficient to fully assess bone health. The current KDIGO guidelines recommend laboratory evaluations of calcium, phosphorus, alkaline phosphatase, and PTH in selected KTRs, based on CKD progression, while BMD evaluation is recommended only in the first 3 months following transplantation in patients with an eGFR greater than 30 ml/min/1.73 m2 if they receive corticosteroids or have risk factors for osteoporosis [48].

The prevalence of osteoporosis in Italian transplant centers seems to exceed that reported in the literature, with more than half of the centers estimating a prevalence greater than 50%. Conversely, the prevalence of fractures is likely underestimated, as most centers practice routine BMD screening through DEXA scan but do not routinely perform spinal radiography to detect vertebral deformities. However, the estimates of osteoporosis prevalence among KTRs provided by the participants could actually reflect a broader concept of bone fragility rather than densitometric osteoporosis alone.

Approaches to primary fracture prevention vary widely among Italian centers. Although this survey did not address specific immunosuppressive regimens or glucocorticoid doses, many centers do not prescribe preventive treatment for fractures in all patients undergoing chronic glucocorticoid therapy at risk for bone fractures, limiting such therapies only to those with pathological DEXA scan results. A more thorough clinical evaluation and focused clinical history regarding bone disease and fracture history, along with simple vertebral morphometry assessments via X-ray (regardless of BMD or glomerular filtration rate) and bone turnover markers, could help identify patients at risk for fractures and guide further diagnostic and therapeutic interventions. However, these diagnostic tests should be tailored based on the patient’s history, clinical data, and the results of available fracture risk calculators.

Thyroid disorders are common among patients with ESKD and KTRs, but it remains unclear whether their prevalence is higher in KTRs than in the general population or to what extent thyroid dysfunction persists after transplantation. While pre-transplant hypothyroidism, particularly low T3 levels, has been suggested as a potential marker for reduced graft survival, there is limited evidence on the impact of thyroid function on transplant success [49–51]. The KDIGO guidelines do not recommend the routine evaluation of thyroid hormone levels in either kidney transplant candidates or KTRs [52, 53].

However, alterations in thyroid hormone levels after transplantation are often linked to immunosuppressive treatment, especially during the early transplant phase when glucocorticoid doses are higher [54]. A low T3 syndrome and subclinical hypothyroidism are frequently observed in KTRs [55–58]. Low T3 levels correlate with factors like serum creatinine, hemoglobin, and BMI and may help predict graft function, especially within the first 5 years post-transplant [10, 49, 50].

Interestingly, many Italian transplant centers appear to be interested in the thyroid history of both waiting list candidates and KTRs, routinely assessing for thyroid disorders and including thyroid hormone levels as part of the clinical evaluation. However, the timing of these evaluations varies widely among centers. Despite the lack of routine ultrasound screening for thyroid disorders, most centers conduct thyroid ultrasounds for KTRs, albeit inconsistently regarding timing.

Multicenter studies with shared datasets on thyroid disorders could support the development of a standardized approach to diagnosis and treatment across Italy, ensuring more equitable care for KTRs.

Regarding other endocrine disorders, the prevalence of hyperprolactinemia, pituitary adenomas, and acromegaly in KTRs is not well documented in the literature. Hyperprolactinemia, which is associated with CKD, often improves or normalizes post-transplantation [59, 60]. It may contribute to hypogonadism, affect fertility, and negatively influence metabolic health and cardiovascular risk. Although there is a lack of routine prolactin level testing in KTRs (93% of centers do not perform periodic evaluations), there may be a role for more attentive clinical evaluations to identify signs of hyperprolactinemia and its complications early.

Adrenal insufficiency is a main concern for KTRs. Long-term steroid treatment can suppress the hypothalamic-pituitary-adrenal axis, potentially leading to life-threatening adrenal insufficiency during times of physical stress [5, 28, 29, 61]. The KDIGO guidelines recommend withdrawing corticosteroids in low-immunological-risk patients during the first week post-transplant [53]. However, attention to adrenal disorders appears to be quite scarce among the participating transplant centers and adrenal function is not routinely assessed during follow-up in a substantial number of centers, with only 14% conducting regular biochemical evaluations.

More attention should be given to adrenal iatrogenic insufficiency, along with ensuring that patients receive adequate education to recognize early signs of a potential adrenal crisis.

This highlights the need for increased awareness and sensitivity to adrenal insufficiency risks, especially given the recent 2024 guidelines from endocrine societies on glucocorticoid-induced adrenal insufficiency [62].

Hypogonadism, often seen in patients with ESKD, can persist post-transplant, particularly in men, where low testosterone and elevated FSH/LH levels may affect fertility. Women may also experience menstrual irregularities due to immunosuppressive drugs and hypothalamic–pituitary–ovarian axis dysregulation [63]. However, many Italian centers show insufficient interest in investigating gonadal function, with 20% not addressing it during clinical history taking and 35% unaware of the incidence of menstrual irregularities or erectile dysfunction in their KTR populations.

Finally, while kidney transplantation has improved the survival rates for patients with ESKD, quality of life remains a critical issue. Although some surveys have examined KTRs’ psychosocial health and return to work [64], there is limited evidence on how endocrine disorders, including sexuality and fertility concerns, impact their quality of life. Despite recognizing the importance of sexual health, approximately 34% and 38% of Italian transplant centers do not address issues related to sexuality and fertility, respectively, during clinical history taking, and many are unaware of the prevalence of sexual dysfunction in KTRs.

A key limitation of this survey is that, due to the anonymity of the responses, we were unable to analyze potential local or geographical disparities in the management of endocrine disorders across transplant centers.

## Conclusions

The survey revealed significant variability in the management of endocrine disorders across the Italian transplant centers. While some centers routinely assess endocrine health in KTRs, others lack dedicated services and consistent screening practices.

In our opinion, adrenal insufficiency should be considered the primary endocrine concern in KTRs. This is a potentially life-threatening condition, particularly due to the risk of intercurrent illness and suppression of the adrenocortical axis by exogenous glucocorticoids. Bone fragility is also a significant follow-up issue in KTRs, given its association with an increased risk of fractures and the consequent impact on morbidity and mortality. Finally, although often underprioritized, reproductive and sexual health are important management aspects, playing a critical role in patients’ physical and psychological well-being and overall quality of life. A major oversight is that many centers do not routinely assess adrenal function, hyperprolactinemia, pituitary, or gonadal health, highlighting the need for greater awareness and a more standardized approach. A thorough anamnestic evaluation and review of clinical data should be considered essential to identify populations at higher risk for endocrine disorders which may warrant more frequent and personalized assessments. Furthermore, a cost-benefit analysis is necessary to establish a standardized approach to potential laboratory and instrumental screening for endocrine disorders in transplant recipients. Improving the screening and treatment protocols for these conditions could enhance the quality of life and long-term health outcomes for KTRs.

## Supplementary Information

Below is the link to the electronic supplementary material.

Supplementary Material 1
