# Supplementary material for: Awareness of post-transplant endocrine disorders among kidney transplant clinicians: results of an Italian survey
Source: J Endocrinol Invest. 2025 Oct 29;49(3):585–98. doi: 10.1007/s40618-025-02741-y (PMC13018077; doi:10.1007/s40618-025-02741-y)
Supplement: Supplementary file 1 — Supplementary Material 1 [file 40618_2025_2741_MOESM1_ESM.pdf]

QUESTIONNAIRE

GENERAL SECTION (S.1)

1. How many KTRs per year are followed up in your center?

- 50-100
- 100-150
- >150

2. How many years have you been involved in the kidney transplantation program?

- 2-5
- 5-10
- 10-20
- >20

3. At your transplant center, do kidney transplant recipients routinely undergo endocrinological evaluation?

- Yes
- Generally no

4. At your transplant center, do you have the possibility to refer patients to an endocrinology specialist?

- Yes, to a specialistic service of endocrinology in my transplant center NOT dedicated to KTRs
- Yes, to a specialistic service of endocrinology in my transplant center dedicated to KTRs
- No, I send the patient to other structures/hospitals

SPECIFIC SECTIONS

Bone disease (S.2)

5. According to your experience, how many KTRs in follow-up at your center suffer from osteoporosis?

- 5-25%
- 25-50%
- 50-75%
- >75%
- I don't know

6. According to your experience, how many KTRs in follow-up at your center had vertebral or femoral fractures in the past year?

- <5%
- 5-25%
- I don't know

7. During clinical history taking, do you investigate bone diseases in KTRs?

- Yes, sporadically
- Yes, routinely

8. During clinical history taking, how many follow-up patients do you ask about a history of fractures?

- Only patients diagnosed with osteoporosis
- All patients
- None

9. Do you routinely request an evaluation of PTH levels in KTRs with no history of parathyroid disorders?

- Yes, every 6 months
- Yes, annually

10. Do you routinely request vitamin D level evaluation for KTRs?

- Yes, sporadically
- Yes, every 6 months
- Yes, annually

11. Do you routinely request evaluation of bone remodeling biomarkers in KTRs (e.g., Alkaline Phosphatase, CTX, P1NP)?

- Yes, sporadically
- Yes, every 6 months
- Yes, annually
- No

12. Do you routinely request a DEXA scan for KTRs?

- Only if fracture risk factors are present
- Yes, annually
- Yes, every 2 years
- No

13. Do you routinely request thoracic and lumbar spine X-rays for KTRs?

- Only if fracture risk factors are present
- Yes, every 2 years
- No

14. Do you prescribe medications for the primary prevention of fractures in patients chronically exposed to glucocorticoids after transplantation?

- Yes, but only in cases of pathological DEXA results
- Yes, to every patient, regardless of DEXA results
- No, I refer patients to another specialist for this treatment
- No, I prescribe treatment to prevent fractures only as secondary prevention (i.e., in patients with a history of fractures)

15. ONLY IF your answer to " Do you prescribe medications for the primary prevention of fractures in patients chronically exposed to glucocorticoids after transplantation?" was "No, I refer patients to another specialist for this treatment": which specialist?

- Endocrinology specialist
- Rheumatology specialist

Thyroid disorders (S.3)

16. How many KTRs in follow-up at your center developed hypothyroidism during the past year?

- <5%
- 5-25%
- I don't know

17. How many KTRs in follow-up at your center developed hyperthyroidism during the past year?

- None
- <5
- 5-10
- I don't know

18. How many KTRs in follow-up at your center were diagnosed with differentiated thyroid carcinoma during the past year?

- None
- <5
- I don't know

19. How many KTRs in follow-up at your center were diagnosed with medullary thyroid carcinoma during the past year?

- None
- <5
- I don't know

20. During clinical history taking, do you screen for thyroid disorders in KTRs?

- Yes, sporadically
- Yes, routinely
- No

21. Do you routinely request thyroid hormones evaluation for KTRs without a history of thyroid disorders?

- Yes, every 6 months
- Yes, annually
- Yes, every 2 years
- No

22. Do you routinely request thyroid ultrasounds for KTRs without a history of thyroid disorders?

- Yes, annually
- Yes, every 2 years
- No

Pituitary disorders (S.4)

23. How many KTRs in follow-up at your center were diagnosed with single or multiple pituitary hormone deficiencies during the past year?

- None
- <5
- I don't know

24. How many KTRs in follow-up at your center were diagnosed with pituitary adenoma during the past year?

- None
- <5
- I don't know

25. How many KTRs in follow-up at your center were diagnosed with hyperprolactinemia during the past year?

- None
- <5
- I don't know

26. How many KTRs in follow-up at your center were diagnosed with acromegaly during the past year?

- None
- <5
- I don't know

27. During clinical history taking, do you screen for pituitary disorders in KTRs?

- Yes, sporadically
- Yes, routinely
- No

28. Do you routinely request pituitary hormone evaluation for KTRs without a history of pituitary disorders?

- Yes, annually
- No

Adrenal disorders (S.5)

29. How many KTRs in follow-up at your center were diagnosed with adrenal insufficiency during the past year?

- None
- <5
- 5-25
- I don't know

30. How many KTRs in follow-up at your center were diagnosed with adrenocortical adenoma during the past year?

- None
- <5
- 5-25
- I don't know

31. How many KTRs in follow-up at your center were diagnosed with adrenocortical carcinoma during the past year?

- None
- <5
- I don't know

32. How many KTRs in follow-up at your center were diagnosed with pheocromocitoma during the past year?

- None
- <5
- I don't know

33. During clinical history taking, do you screen adrenal disorders in KTRs?

- Yes, sporadically
- Yes, routinely
- No

34. Do you routinely request an evaluation of adrenal hormones for KTRs without a history of adrenal disorders?

- Yes, every 2 years
- Yes, annually
- No

Gonadal disorders, fertility, and sexuality (S.6)

35. How many KTRs in follow-up at your center developed hypogonadism during the past year?

- <5%
- 5-25%
- I don't know

36. How many KTRs in follow-up at your center developed erectile dysfunction during the past year?

- <5%
- 5-25%
- 25-50%
- I don't know

37. How many KTRs in follow-up at your center developed low libido during the past year?

- <5%
- 5-25%
- 25-50%
- 50-75%
- I don't know

38. How many KTRs in follow-up at your center developed menstrual irregularity during the past year?

- <5%
- 5-25%
- 25-50%
- 50-75%
- I don't know

39. How many KTRs in follow-up at your center developed clinical/biochemical hyperandrogenism during the past year?

- <5%
- 5-25%
- I don't know

40. During clinical history taking, do you screen for gonadal disorders in KTRs?

- Yes, sporadically
- Yes, routinely
- No

41. During clinical history taking, do you assess sexual health in KTRs?

- Yes, sporadically
- Yes, routinely
- No

42. During clinical history taking, do you assess the fertility status of KTRs?

- Yes, sporadically
- Yes, routinely
- No

43. During clinical history taking, do you assess menstrual regularity of KTRs?

- Yes, sporadically

- Yes, routinely
- No

44. Do you routinely request an evaluation of gonadal hormones for KTRs without a history of gonadal disorders?

- Yes, every 6 months
- Yes, annually
- Yes, every 2 years
- No
